# The Role of the Different Components of Attention on Observational Learning in Early Primary School Children: New Insights and Educational Implications

**DOI:** 10.3390/brainsci16020237

**Published:** 2026-02-19

**Authors:** Francesca Foti, Valentina Lucia La Rosa, Luca Pullano, Tiziana Iaquinta, Elena Commodari

**Affiliations:** 1 Department of Educational Sciences, University of Catania, 95124 Catania, Italy; valentina.larosa@unict.it; 2 Department of Medical and Surgical Sciences, “Magna Graecia” University of Catanzaro, 88100 Catanzaro, Italy; luca.pullano@unicz.it

**Keywords:** observational learning, learning by observation, imitation, social learning, learning, attention, early primary school, cognitive development

## Abstract

**Highlights:**

**What are the main findings?**

- Visual and visual–spatial focused attention significantly contribute to observational learning.
- Reaction time related to a choice shows different associations across observational learning phases.

**What are the implications of the main findings?**

- Designing instructional contexts that support attentional engagement may improve children’s observation learning.
- Differentiated practices and visual–spatial activities may enhance children’s observational learning.

**Abstract:**

**Background/Objectives:** Observational learning enables children to acquire new skills by observing others’ actions. Attention is widely recognized as a key supporting process and consists of multiple components that develop substantially during the early school years. Empirical evidence on the association between specific components of attention and observational learning remains limited. Therefore, this study examined the relationship between the main components of attention and observational learning among early primary school children. **Methods:** Sixty-eight children, aged 6–8, completed a computerized battery assessing the main components of attention (reaction times, simple and related to a choice; focused attention; short-term span of attention; divided and alternating attention) and an observational learning task where children observed an actor detecting a hidden spatial sequence and then reproduced it across detection phase (DP), exercise phase (EP), and automatization phase (AP). Correlational and regression analyses were conducted, controlling for age and gender. **Results:** Visual and visual–spatial focused attention emerged as significant predictors of performance during DP and EP, with higher levels of focused attention associated with fewer errors and repetitions. Choice reaction time showed phase-specific associations with error rates during early learning phases, whereas age was primarily related to performance during the AP. **Conclusions:** Observational learning in early primary school relies on specific components of attention rather than on attention as a unitary construct. Visual and visual–spatial focused attention plays a central role during the acquisition and consolidation of observed sequences, with implications for understanding learning from models and for educational practices based on demonstration.

## 1. Introduction

Humans can acquire new skills either through direct experience (*learning by trial and error* or *learning by doing*) or by observing the actions of others (*observational learning*) [1,2]. Observing another person performing a complex action or solving a problem facilitates the observer’s acquisition of the same behavior, shortening the time needed to learn through trial and error and reducing the number of attempts required [2,3]. According to Bandura’s social cognitive theory, observational learning does not merely involve copying or mechanically imitating an action. Rather, it requires the observer to transform the observed behavior into an action that matches the model’s goal and motor strategies [4,5,6,7]. For this reason, observational learning involves multiple cognitive processes, including action representation, attention, and motivation. It also requires the ability to interpret others’ gestures, infer intentions, and process social information [6,8]. Specifically, according to the classical theoretical framework of Bandura’s social cognitive theory [1,9], observational learning is characterized by four phases, each involving specific cognitive and affective mechanisms: attentional processes, retention processes, motoric reproduction processes, and reinforcement and motivational processes. Attentional processes are necessary because learning occurs only when the observer focuses on the model’s relevant behaviors. Retention processes allow observed actions to be encoded and stored in memory so they can be autonomously reproduced. Motoric reproduction processes translate these internal representations into actual behaviors. Finally, reinforcement and motivational processes determine whether the learned behavior is enacted, but also how frequently it is performed, depending on whether it is encouraged or discouraged. Moreover, observational learning is already present at birth and develops gradually [2,10,11,12]. It represents a powerful mechanism of social learning that allows information to be transferred between individuals and supports adaptive functioning [13]. During development, observation is also involved in acquiring academic skills [14] and can be an effective instructional approach in reading, writing, and other academic competencies [15]. Interestingly, recent studies have demonstrated that observation also promotes an earlier acquisition of high-level spatial skills in typically developed children [16] and is a powerful way to facilitate the acquisition of complex behaviours also in neurodevelopmental disorders such as Williams or Prader–Willi syndromes, as well as autism spectrum disorder [17,18,19,20].

Among the cognitive processes that support observational learning, it is widely recognized that attention plays a pivotal role. Indeed, the ability to focus on relevant aspects of the model’s behavior, maintain concentration throughout the observation phase, and filter out distractions determines how effectively the observed action is encoded, represented, and later reproduced [21,22]. In this sense, attention functions as a “gatekeeper” of learning, regulating which elements of experience are selected, processed, and integrated into long-term memory. Within observational settings, attentional mechanisms guide the allocation of cognitive resources toward salient or goal-related cues, enhancing subsequent imitation and problem-solving performance [23].

However, attention is not a unitary process, but a complex, multi-component system that has been extensively described within cognitive and developmental frameworks as comprising distinct yet interacting processes [24,25,26,27,28]. During childhood, these components develop progressively and follow differentiated developmental trajectories [29]. This is particularly evident during early school age, when attentional regulation and executive control undergo substantial refinement [30]. Within these frameworks, attentional functioning includes specific components such as focused attention, divided attention, and alternating attention, each supporting distinct aspects of goal-directed behavior. *Focused attention* refers to the ability to allocate attentional resources on task-relevant stimuli while managing competing or distracting information, thereby supporting efficient stimulus detection and processing [31]; *divided attention* allows the simultaneous processing of multiple input streams; *alternating attention* enables flexible shifting between tasks or mental sets [25,27,32,33,34,35]. Moreover, at its most basic level, early attentional functioning includes processes related to alertness and physiological activation, which enable a child’s readiness to respond to environmental stimuli. These processes are typically assessed using simple reaction time tasks and form the foundation upon which more complex regulatory processes are built [30,31,35]. Furthermore, an adequate functioning of these components requires the ability to temporarily hold and manage information, *short-term span of attention*, which supports the maintenance, updating, and coordination of attentional resources during goal-directed activity [36,37]. Finally, these components are supported by distinct yet interconnected neural networks (*alerting*, *orienting*, and *executive control*) that together sustain goal-directed behavior [29,38,39]. It is important to note that these components are not expected to contribute uniformly to different learning demands. Rather, they may play distinct roles depending on the characteristics of the task and the learning phase.

The early school years represent a particularly sensitive period for the refinement of attentional control [40,41]. The transition from preschool to the first years of primary school (ages 6–8) marks a developmental turning point characterized by increased self-regulation, reduced distractibility, and greater flexibility in monitoring and adjusting behavior [35,42,43]. These advances reflect the maturation of frontal and parietal brain regions, which support the integration of executive and orienting attentional systems [44]. At the same time, the school environment itself places growing demands on attentional resources: children are required to remain focused for longer periods, filter competing sensory inputs, and extract task-relevant information from complex visual and auditory settings [45].

In this developmental window, attentional processes become critical not only for academic learning but also for social and observational learning. Observing others’ behaviors, grasping their intentions, and translating these into one’s own actions depend on the coordinated functioning of selective and executive attentional systems. Furthermore, the focus on attentional processes is theoretically motivated by converging evidence indicating that attention plays a foundational role in higher-order cognitive operations by guiding the selection of environmental information for further processing, including working memory encoding and long-term memory consolidation [46,47]. Within observational learning contexts, attention represents a functionally prior process that conditions the effectiveness of subsequent stages, such as retention and motoric reproduction. Although observational learning involves multiple cognitive and motivational components [9,48], focusing on attention allows for a principled examination of the initial mechanisms through which observed information is encoded, organized, and transformed into learning outcomes.

Nevertheless, despite the centrality of attention in shaping observational learning processes, relatively few and largely dated studies have examined how multiple components of attention jointly and differentially contribute to observational learning during early schooling, and even fewer have considered observational learning as a multi-phase process rather than as a single outcome [49,50,51,52]. Moreover, previous research has rarely addressed how specific components of attention relate to the types and frequency of errors children make during observational learning, limiting our understanding of the cognitive mechanisms underlying learning failures and inefficiencies. As a result, it remains unclear whether the same attentional components support early acquisition, practice, and later automatization of observed behaviors, as well as how attentional functioning shapes error patterns across these phases. To address this gap, the present study investigates the relationship between the main components of attention and the specific phases of observational learning in children attending the first two grades of primary school. Specifically, the study aims to examine the role of specific components of attention across distinct phases of observational learning (detection, exercise, and automatization), as well as in relation to the occurrence and types of errors produced during task performance. This approach aims to provide a deeper understanding of how attentional functioning supports not only successful learning from observation but also learning-related errors during a critical period of cognitive development.

The results of the present study are the first of this type and could provide a more precise characterization of observational learning by analyzing its underlying components of attention. Such characterization could enhance the effectiveness of observational learning across different contexts. In the educational context, identifying which components of attention are involved may help teachers design tasks and demonstrations that are better aligned with children’s heterogeneous attentional profiles. This, in turn, may improve the efficiency of learning through observation.

Moreover, clarifying the components of attention involved in observational learning may allow these same paradigms to be used as tools to strengthen attention itself. This dual benefit—using attention to improve observational learning and using observational learning to support attentional development—could be beneficial not only for typically developed children but also for clinical populations with attentional difficulties.

Beyond educational contexts, a clearer understanding of how attention shapes observational learning could also be useful for everyday social interactions, supporting the acquisition of norms, routines, and adaptive behaviors.

### Research Questions

In line with this theoretical and empirical background, the present study was guided by the following research questions:

1. Do different components of attention show differential associations with observational learning performance during early primary school age?
2. Are specific components of attention differentially related to distinct phases of observational learning (detection, exercise, and automatization), reflecting the dynamic nature of the learning process?
3. How are different components of attention associated with the occurrence and types of errors produced during observational learning?

## 2. Materials and Methods

### 2.1. Participants

Sixty-eight children (42 females and 26 males) aged from 5 years and 11 months (5.11) to 8 years and 1 month (8.01) (M = 6.40, SD = 0.60) participated in the present study. Participants were recruited using a non-probabilistic convenience sampling procedure from a single public primary school in southern Italy (Sicily). Inclusion criteria were: (a) typical development; (b) enrollment in the first or second grade of primary school; and (c) provision of valid written informed consent by parents or legal guardians. Children with certified neurodevelopmental, neurological, or psychiatric diagnoses were not included. In the Italian school system, such diagnoses are formally certified according to national regulations governing special educational needs and access to educational support services, and are systematically documented within the school context. Additional exclusion criteria included visual or auditory impairments, or any condition that could interfere with comprehension of the instructions or task performance. Participation was voluntary, and written informed consent was obtained from parents or legal guardians before participation. The study was approved by the Institutional Review Board of the Department of Educational Sciences at the University of Catania (Ierb-Edunict-2024.03.07/02) and conducted in accordance with the principles of the Declaration of Helsinki.

### 2.2. Behavioral Tasks and Measures

#### 2.2.1. Attention and Concentration Battery

**Figure 1:**
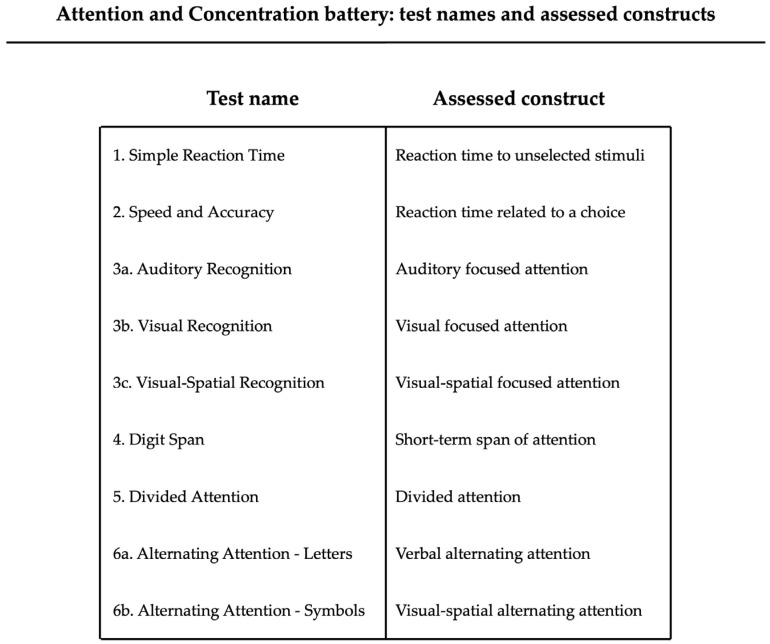
Schematic representation of the Attention and Concentration Battery.

Specific components of attention were assessed using the Attention and Concentration Battery [53,54], a computerized instrument validated across different age groups. The battery includes age-specific task levels, with difficulty calibrated to the child’s developmental stage. For the present study, the version designed for early and middle childhood was administered. The battery comprises a set of tasks that jointly evaluate multiple components of attention, including reaction time to unselected stimuli; reaction time related to a choice; auditory, visual, and visual–spatial focused attention; short-term span of attention; divided attention; and alternating attention (Figure 1).

The first test, “Simple Reaction Time”, measures reaction time in response to a neutral visual stimulus, requiring children to press a key as soon as a briefly appearing star is displayed on the screen. The second test, “Speed and Accuracy”, assesses reaction time related to a choice through numerical stimuli: sequences of digits from 1 to 6 are presented, and the child must press the key corresponding to the number highlighted in red. The third test consists of three subtests that assess focused attention in auditory, visual, and visual–spatial modalities: the “Auditory Recognition” subtest measures auditory focused attention and participants are asked to respond whenever they hear the vowel “o” among a sequence of spoken letters; the “Visual Recognition” subtest measures visual focused attention and participants must press a key each time a specific target image appears among a series of familiar pictures; the “Visual–Spatial Recognition” subtest measures visual–spatial focused attention and consists of a computerized barrage test in which a matrix of 36 figures is displayed; each figure is sequentially outlined on the screen, and the child must identify and delete the target symbol as quickly and accurately as possible. The fourth test, “Digit Span”, assesses short-term span of attention. It is a computerized adaptation of the classic Digit Span from the Wechsler Scale [55], which includes forward and backward conditions requiring children to repeat a sequence of digits in the same or reverse order. The fifth test, “Divided attention”, evaluates divided attention through a dual-task paradigm combining concurrent visual and auditory recognition tasks: children respond simultaneously to a visual target appearing among distractor images and to an auditory target embedded within a stream of spoken words. Finally, the sixth test, “Alternating Attention”, assesses the ability to shift attentional focus between changing sets of stimuli through a computerized nonverbal cancellation paradigm. This test includes both verbal (letters) and visual–spatial (symbols) subtasks, modeled on the classic Toulouse–Piéron test [56], requiring rapid adaptation of attentional strategies.

Stimulus presentation and data recording were fully automated, ensuring standardization across participants. Previous research has demonstrated strong psychometric properties for the battery, with test–retest reliability coefficients ranging from 0.82 to 0.92 and concurrent validity indices between 0.80 and 0.90.

*Parameters*. For each test, the number of correct answers and the Response Times (RTs; in seconds) were calculated, except for the Digit Span test, for which a total score was calculated by adding the number of digits correctly remembered in the forward and backward conditions.

#### 2.2.2. Observational Learning Task

The observational learning task applied in the present study was used and described in detail in several previous studies [17,18,19].

**Figure 2:**
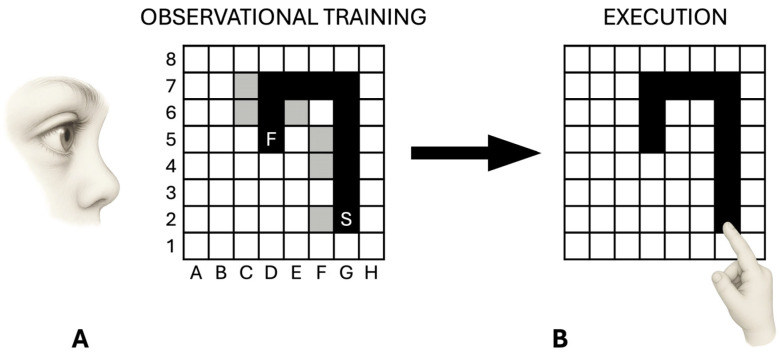
Schematic representation of the observational learning task. Following the observational training phase (**A**), participants reproduced the observed sequence autonomously (**B**). The black squares indicate the correct sequence to be learned, whereas the grey squares represent the six incorrect positions deliberately touched by the actor during the observational training. “S” indicates the starting point and “F” the final point.

Children sat in front of a computer touch screen on which an 8 × 8 black matrix appeared. Children were required to detect a hidden sequence of 10 correct squares forming a path (Figure 2). To explain the task, the experimenter used the following verbal instruction: “You have to find a path formed by ten squares. When you touch a correct square belonging to the path, it will be turned grey and you will hear a sound; conversely, if you touch a wrong square not belonging to the path, it will be turned red. In this case, you have to find a new grey square. You have to restart each time you find a new correct square. After finding the whole path, you have to retouch it three times without errors”. The participants started by touching a grey square, which was the first element of the sequence and was always lit up. In the search for the second correct square, the participants had to touch one of the four squares bordering the grey square by moving in the matrix vertically or horizontally, but never diagonally. Each touched square (correct or wrong) was lit up for 500 ms and then the light went off again; thus, no trace of the touched sequence remained on the screen.

After the verbal instructions, children observed the experimenter acting as an actor, detecting the entire sequence by trial and error (observational training). In performing the task, the actor always made the same six errors, so that all participants observed the same pattern of correct and wrong touches (Figure 2A). After the observational training, children were asked to autonomously reproduce the observed sequence (Figure 2B).

*Parameters.* The observational learning task involved three phases: the Detection Phase (DP), which finished once children found the tenth correct position; the Exercise Phase (EP), in which children repeated the sequence until their performance was error-free; and the Automatization Phase (AP), which finished when the correct sequence was repeated three consecutive times without errors.

Since the AP phase encompassed three consecutive error-free trials, if—for example—after two consecutive error-free trials during the third trial an error was performed, the entire triplet of error-free trials had to be repeated.

The parameters measured were: *DP errors*, the number of incorrect items touched in detecting the 10 correct positions; *EP repetitions*, the number of replications needed to reach an error-free performance; *AP times* (in ms)*,* the time spent carrying out each of the three consecutive error-free repetitions of the sequence.

To further characterize the performance of children, a deep analysis of the errors was performed. Specifically, errors were divided into five types: *perseverative errors,* touching the same square or a fixed sequence of squares; *sequence errors*, touching a correct square at the wrong moment (e.g., touching D7 before E7); *side-by-side errors*, touching the squares bordering the correct sequence (e.g., H5); *illogical errors*, touching any other square (e.g., A8); *imitative errors*, touching the square deliberately wrongly touched by the actor during the observational training (e.g., F5).

### 2.3. Procedure

Children were tested individually in a dedicated classroom within their school during regular school hours. Each session lasted approximately 50 min and was divided into two main parts: administration of (1) the Attention and Concentration Battery and (2) the Observational Learning Task.

The order of the tasks was counterbalanced across participants; a short break was planned between tasks. Each child was first familiarized with the computer interface and response keys. The attentional tasks were introduced through brief practice trials to ensure comprehension of instructions. The observational learning task was presented following the verbal instructions described above.

### 2.4. Statistical Analyses

All statistical analyses were conducted using the R statistical programming environment [57] and Jamovi [58]. Following the approach adopted in previous studies employing the Attention and Concentration Battery [33,59,60], a series of partial correlations and multiple linear regression models—using the “entry forced” method—was conducted in order to examine the relationships between attention components and variables of the observational learning task (the three parameters related to the three phases as well as the five types of errors). Prior to model estimation, multicollinearity diagnostics were performed, including the calculation of variance inflation factors (VIFs), which did not indicate problematic levels of collinearity among predictors.

Particularly, in the first set of analyses, we explored the contribution of components of attention to the observational learning phases. For the Attention and Concentration Battery, we considered the number of correct responses and the RTs for each subtest, except the *Digit Span*, in which the total score was obtained by summing the span from forward and backward conditions. Partial correlations with age and gender as covariates were first conducted to preliminarily assess the associations between measures of attention and the parameters of the observational learning phases (i.e., *DP errors*, *EP repetitions*, and *AP time*), controlling for age and gender. Subsequently, multiple linear regression models were computed to identify whether age, gender, and measures of attention (independent variables) predicted performance in each phase of the observational learning task (dependent variables). The second set of analyses followed the same procedure, this time focusing on the parameters related to the five types of errors performed during the observational learning task (i.e., *perseverative, sequence, side-by-side, illogical, and imitative errors*).

## 3. Results

Descriptive statistics for all variables of the Attention and Concentration Battery and Observational Learning Task are reported in Table 1. To improve clarity, the following sections describe the statistical analyses divided into the first and second sets, as described above.

### 3.1. First Set of Analyses—Observational Learning Task Phases

In Table 2, the coefficients of partial correlation with age and gender as covariates are reported, corrected for multiple comparisons. Overall, several significant associations were observed between measures of attention and performance across the phases of the observational learning task. Specifically, *DP errors* were negatively correlated with *Digit Span* score (*r* = −0.47, *p* < 0.05); *EP repetitions* were positively correlated with the RTs in the *Speed and Accuracy* test (*r* = 0.65, *p* < 0.001), and negatively correlated with correct responses in the *Speed and Accuracy* test (*r* = −0.59, *p* < 0.001) and in the *Visual–Spatial Recognition* test (*r* = −0.67, *p* < 0.001). No significant correlations were observed between *AP time* and measures of attention.

Table 3 presents the multiple linear regression analyses conducted to examine the contribution of age, gender, and measures of attention to performance across the three phases of the observational learning task.

For *DP errors,* the entry forced model explained approximately 46% of the variance (*F*_(19,48)_ = 2.18, *R*^2^ *=* 0.46, *p* = 0.01). Specifically, RTs in the *Speed and Accuracy* test (*B* = −0.42, *t* = −2.25, *p =* 0.03) and correct responses in the *Visual–Spatial Recognition* test *(B* = −0.41, *t* = −2.04, *p =* 0.04) were significant predictors. Thus, higher RT_S_ in the *Speed and Accuracy* test and more correct responses in the *Visual–Spatial Recognition* subtest were associated with fewer errors performed during the *Detection Phase*.

For *EP repetitions*, the entry forced model accounted for approximately 66% of the variance (*F*_(19,48)_ = 4.91, *R*^2^ *=* 0.66, *p* < 0.001). RTs in the *Speed and Accuracy* test (*B* = 0.31, *t* = 2.13, *p* = 0.04) and correct responses in the *Visual–Spatial Recognition* test (*B* = −0.58, *t* = −3.67, *p* < 0.001) emerged as significant predictors. Thus, higher RTs in the *Speed and Accuracy* test were associated with a higher number of repetitions, while more correct responses in the *Visual–Spatial Recognition* test were associated with fewer repetitions needed to reach error-free performance in the observational learning task.

For *AP time*, the entry forced model was not significant (*F*_(19,48)_ = 1.09, *R*^2^ *=* 0.30, *p* = 0.40), indicating that the model did not account for a substantial proportion of variance in the criterion. However, the independent variable *Age* (*B* = −0.31, *t* = −2.02, *p* = 0.04) showed significant effects.

### 3.2. Second Set of Analyses—Observational Learning Task Errors

Regarding the five types of errors performed during the observational learning task, Table 4 reports the coefficients of partial correlation with age and gender as covariates. Overall, several significant correlations were observed between measures of attention and specific error types performed during the observational learning task.

*Sequence errors* were negatively correlated with correct responses in the *Visual–Spatial Recognition* test (*r* = −0.59, *p* < 0.001), and positive correlated with the RTs in the *Alternating Attention–Symbols* test (*r* = 0.42, *p* < 0.05). *Side-by-side errors* were negatively correlated with the correct responses in the *Visual–Spatial Recognition* test *(r* = −0.44; *p* < 0.05). *Illogical errors* were positively correlated with RTs in the *Alternating Attention–Symbols* test (*r* = 0.46, *p* < 0.05). *Imitative errors* were negatively correlated with correct responses in the *Speed and Accuracy* test (*r* = −0.46, *p* < 0.001) and in the *Visual–Spatial Recognition* test (*r* = −0.61, *p* < 0.001) and positively correlated with RTs in the *Alternating Attention–Symbols* test (*r* = 0.46, *p* < 0.001). No significant correlations were found between measures of attention and *perseverative errors.*

Table 5 presents the multiple linear regression analyses conducted to examine the contribution of age, gender, and measures of attention to the different types of errors performed during the observational learning task.

For *perseverative errors*, the entry forced model explained approximately 45% of the variance (*F*_(19,48)_ = 2.07, *R*^2^ *=* 0.45, *p* = 0.02). Within this model, correct responses in the *Visual–Spatial Recognition* test (*B* = −0.44, *t* = −2.16, *p* = 0.03) and in the *Alternating Attention–Symbols* test (*B* = −0.26, *t* = −2.01, *p* = 0.04) emerged as significant predictors. Thus, more correct responses in the *Visual–Spatial Recognition* and *Alternating Attention–Symbols* tests were associated with fewer *perseverative errors*.

For *sequence errors*, the entry forced model accounted for approximately 54% of the variance (*F*_(19,48)_ = 2.98, *R*^2^ = 0.54, *p* = 0.001). Correct responses in the *Visual–Spatial Recognition* test (*B* = −0.65, *t* = −3.51, *p* < 0.001) were the only significant predictors. Thus, more correct responses were associated with lower *sequence errors* performed in the observational learning task.

For *side-by-side errors*, the entry forced model explained approximately 43% of the variance (*F*_(19,48)_ = 1.91, *R*^2^ = 0.43, *p* = 0.04). Correct responses in the *Visual Recognition* test (*B* = −0.47, *t* = −2.55, *p* = 0.01) and in the *Visual–Spatial Recognition* test (*B* = −0.51, *t* = −2.49, *p* = 0.02) were significant predictors. Therefore, more correct responses in both tasks were associated with lower *side-by-side* errors performed in the observational learning task.

For *illogical errors*, the entry forced model was not statistically significant (*F*_(19,48)_ = 1.61, *R*^2^ = 0.39, *p* = 0.09), indicating that the model did not account for a substantial proportion of variance in the criterion. *Age* (*B* = 0.37, *t* = 2.57, *p* = 0.01) and RTs in the *Alternating Attention–Symbols* test (*B* = 0.42, *t* = 2.23, *p* = 0.03) showed individually significant effects.

For *imitative errors*, the entry forced model was statistically significant and accounted for approximately 56% of the variance (*F*_(19, 48)_ = 3.24, *R^2^* = 0.56, *p* < 0.001). Within this model, correct responses in the *Visual–Spatial Recognition* test emerged as the only significant predictor (*B* = −0.55, *t* = −3.03, *p* = 0.004). Therefore, more correct responses in the *Visual–Spatial Recognition* task were associated with lower *imitative errors* performed in the observational learning task.

## 4. Discussion

The present study aimed to investigate the relationships that occur between attention and observational learning. In particular, although the relationship between attention and observational learning is well established at a general level, the present findings contribute to clarify the relationship between the main components of attention (reaction times, both simple and related to a choice, focused attention, short-term span of attention, divided and alternating attention) and observational learning (specifically, phases of the observational learning task: detection, exercise, and automatization phases; and possible errors that occurred during the task: perseverative, sequence, side-by-side, illogical, and imitative errors).

Regarding the observational learning task phases, the results of the correlation analysis highlighted associations with some components of attention. Specifically, a higher short-term span of attention was associated with fewer errors performed during the *detection phase*, i.e., when children—after the observational training—autonomously found the path for the first time by translating the observed information into action. Conversely, the number of repetitions needed during the *exercise phase*—i.e., when children consolidated the information to reach an error-free performance—was associated with lower visual–spatial focused attention and with higher RTs in the *Speed and Accuracy* test, which assesses reaction times related to a choice. Finally, no association emerged between attention components and the *automatization phase*, i.e., when children purely automatized the sequence performed during the previous phases.

However, the multiple linear regression analyses suggested the specific contribution of the components of attention. Correct responses in the *Visual–Spatial Recognition* test, as well as the RTs of the *Speed and Accuracy* test, were significantly related to both the *detection* and *exercise* phases. Conversely, the regression model for the *automatization phase* was not statistically significant, although age emerged as the only variable showing a significant independent association. Overall, these results suggest that visual–spatial focused attention and the rapidity in reaction time related to a choice have an important role in observational learning. Thus, it seems that stronger visual–spatial focused attention may have supported more effective encoding of the observed path. This, in turn, may have reduced errors and the number of repetitions required. This pattern is also coherent with theoretical models of working memory, particularly Baddeley’s framework, according to which visuospatial information is maintained in the visuospatial sketchpad and regulated by the central executive [36]. The observational learning task used in this study requires continuous updating, suppression of interference, and precise maintenance of spatial information, processes that depend on the coordinated functioning of these systems.

Regarding the rapidity in reaction time related to a choice, its contribution differs depending on the specific phase of the observational learning task. Indeed, during the *detection phase*, longer RTs were associated with fewer errors. This pattern may reflect a more deliberate response style in some children, which could have supported more accurate recall and reproduction of the observed sequence. In other words, a less impulsive and more reflective response style might have facilitated the internalization of the sequence learned during the observational training, thereby improving performance during the subsequent autonomous discovery phase. However, this interpretation should be directly tested in future studies. Conversely, in the *exercise phase*, longer RTs were associated with a higher number of repetitions before reaching an error-free performance. This pattern may indicate that slower responding during this phase reflects a less consolidated representation of the sequence, resulting in the need for additional repetitions. Regarding the *automatization phase*, this phase reflects pure automatization of the visuo-motor sequence, relying primarily on procedural memory [61,62]. At this stage, attentional demands may play a reduced role, while performance could be increasingly influenced by age-related factors, such as the progressive refinement of motor control and procedural efficiency.

From the deep analyses of the errors performed in the observational learning task, it emerged that the most common errors were sequence errors, followed by side-by-side and imitative ones. The lower number of perseverative and illogical errors suggests that all participants managed the task fundamentals. In fact, the observational learning—that reduces the errors and the time required to acquire new information—permitted to avoid these types of errors that are more common during other types of learning, such as in learning by trial and error. Moreover, these results are in line with previous studies in which the same observational leaning task was used [17,18,19].

Moving to the partial correlation analyses conducted, the results highlighted significant associations between components of attention and types of errors. In particular, visual–spatial focused attention was negatively correlated with sequence, side-by-side, and imitative errors, whereas reaction time related to a choice was negatively correlated with imitative errors, and finally, the RTs of visual–spatial alternating attention were positively correlated with side-by-side, illogical and imitative errors. No significant associations were found between components of attention and perseverative errors.

Similarly to what was described above, the multiple linear regression analyses suggested the specific contribution of the components of attention. Visual–spatial focused attention was found to be significantly associated with almost all types of errors, except for the illogical ones. Visual focused attention was associated with side-by-side errors. The correct responses of the visual–spatial alternating attention were associated with perseverative errors, while its RTs and age were associated with the illogical errors.

Once again, focused attention emerged as the primary attention component predicting observational learning, especially for sequence, side-by-side, and imitative errors, where no other attention components reached significance. Unlike perseverative and illogical errors, which can be produced by simply touching squares that are not part of the sequence, these three error types are directly linked to the learning process itself and specifically to the observed sequence. Thus, the ability to focus on visual and visual–spatial information, resist distraction, and inhibit a response that was previously correct or observed, but no longer appropriate, may be associated with a lower occurrence of errors that are directly linked to the learning of the observed sequence, allowing a better knowledge of the sequence and, consequently, increasing the efficacy of the observational learning. Hence, the components of attention most strongly involved were the visual and visual–spatial ones, likely due to the nature of the task.

Visual–spatial focused attention, as well as visual–spatial alternating attention, were also significant predictors of perseverative errors. Specifically, a better ability to rapidly shift the attentional focus from one cognitive task to another [63] and the ability to disengage and reengage the focus of attention in response to environmental stimuli [25] seem predictive of committing fewer perseverative errors. In other words, children who were able to efficiently shift their attentional focus across the different demands of the task—remembering the sequence, selecting the correct square, and updating their position within the sequence—performed fewer perseverative errors.

Finally, although the predictive model was not significant for illogical errors, a higher number of illogical errors was associated with older age and longer reaction times in visual–spatial alternating attention. This pattern should be interpreted with caution. One possible explanation is that older children may engage in more exploratory or self-initiated behaviors when interacting with the task, which could temporarily increase the occurrence of responses that deviate from the observed sequence [64,65]. However, the present study did not directly assess exploratory strategies or intentional deviations from the model. Future studies combining observational learning paradigms with direct measures of strategy use, metacognitive monitoring, or exploratory behavior will be essential to clarify the mechanisms underlying age-related differences in error patterns.

Overall, our results suggest that visual and visual–spatial focused attention play a pivotal role in observational learning. Indeed, observational learning requires transforming the observed behavior into an action that is as accurate as possible, minimizing errors and ignoring distracting stimuli [4,5,6]. For this reason, focused attention appears to be the most suitable attention component. Moreover, rapidity in reaction time related to a choice was also associated with observational learning. However, when errors performed in the observational learning task were examined in detail, rapidity in choice reaction time did not emerge as a significant contributor, whereas focused attention remained the most influential factor.

### 4.1. Study Limitations and Future Directions

Despite the present study allows to achieve important new insights on the relationship between distinct components of attention and observational learning, it also presents some limitations that open several opportunities for future studies.

First, the relatively small sample size and the cultural homogeneity of the participants, who were all recruited in Southern Italy, limit the breadth of inference. Nevertheless, it is important to note that the Italian primary school system is characterized by nationally standardized curricula, and access to primary school teaching requires formal university-level training and national qualification. Consequently, core instructional practices and curricular objectives are largely homogeneous across the country, which may reduce variability related to educational exposure within the Italian context. At the same time, these characteristics underscore the need for future cross-cultural studies involving educational systems with different curricular structures, pedagogical traditions, and teacher training pathways, in order to examine the robustness and generalizability of the observed associations across diverse learning environments.

In addition, the age range examined (6.1–8.1 years) corresponds to a sensitive developmental period characterized by rapid changes in attentional control and executive functioning. Although this relatively narrow window was intentionally selected to reduce developmental heterogeneity and to capture individual differences within a comparable stage of cognitive maturation, future research should extend this approach to younger and older age groups to investigate developmental continuity and change in the relations between attention and observational learning.

Regarding observational learning, the present study used a visuomotor task to assess attentional engagement during action observation and reproduction in a controlled manner. Although this paradigm offers strong experimental control and has been widely used in previous research, it captures only a subset of the processes involved in real-world observational learning. Real-world observational learning typically unfolds in multimodal, socially embedded, and linguistically mediated contexts. Therefore, future studies should adopt a broader range of observational paradigms that incorporate different sensory modalities and task characteristics to allow researchers to examine whether distinct components of attention are differentially recruited depending on the ecological demands of the learning situation.

Moreover, although the present study focused specifically on attentional processes, previous research has highlighted the role of other cognitive functions, such as working memory and cognitive flexibility, in supporting observational learning [66,67,68]. Future investigations should therefore examine the combined contribution of multiple executive and cognitive processes to provide a more comprehensive account of the mechanisms underlying observational learning in childhood.

Finally, the observational task used in the present study has been successfully applied in previous research involving clinical populations, including individuals with Down syndrome, Williams syndrome, Prader–Willi syndrome, and autism spectrum disorder [17,18,19]. Replicating the current study in these populations would make it possible to examine whether different neurodevelopmental conditions are associated with distinct patterns of attentional engagement during observational learning. Such evidence could inform the development of targeted educational and clinical interventions aimed at optimizing learning strategies and improving quality of life.

### 4.2. Educational Implications

The findings of this study have meaningful implications for educational practices, especially in the early years of primary school when observational learning is central to instruction. In everyday classroom contexts, children are frequently required to learn new procedures, routines, and problem-solving strategies by observing teacher demonstrations or peer models, rather than through explicit verbal instruction alone. Such activities place substantial demands on children’s ability to allocate attention efficiently, particularly in visually and spatially rich learning environments.

From an educational perspective, these findings highlight the importance of designing instructional contexts that support children’s attentional engagement during observation. Classroom demonstrations that are visually clear, well structured, and free from unnecessary distractors are likely to facilitate more effective learning. Segmenting complex actions into smaller, sequential steps, emphasizing critical spatial relationships, and pacing demonstrations appropriately may help children maintain focus and encode relevant information more accurately. The strategic use of brief verbal cues to direct attention to key elements of the demonstration can further support learning without increasing cognitive load.

These results also highlight the value of adopting differentiated instructional practices that take into account individual variability in attentional capacities, even within typically developing populations. Children differ markedly in how efficiently they focus attention, and these differences could influence how they benefit from observational learning opportunities. Teachers who are sensitive to such variability may be better positioned to adapt their instructional strategies, for example by providing additional demonstrations, allowing extra observation time, or offering guided practice following observation.

Finally, incorporating classroom activities that promote visual–spatial attention may indirectly enhance children’s ability to learn through observation. Games and learning tasks that require visual attention, spatial tracking, or the inhibition of irrelevant stimuli can strengthen attentional control and promote more efficient engagement with instructional models. In this way, supporting attentional development within the classroom may contribute not only to improved academic performance, but also to more effective learning from social and instructional contexts that rely on observation.

## 5. Conclusions

In conclusion, the present study provides new evidence of the pivotal role of attentional processes in supporting observational learning during the early primary school years. Specifically, visual and visual–spatial focused attention were the strongest predictors of children’s ability to accurately encode, retain, and reproduce the observed sequence. Rapidity in the task that measures the reaction time related to a choice also contributed to performance, though its influence varied across learning phases, underscoring the dynamic interplay between deliberate processing and emerging automatization. These findings suggest that observational learning relies on distinct components of attention.

Taken together, this work reinforces the idea that attentional skills are fundamental to children’s ability to learn from others, offering theoretical and practical insights for designing effective learning environments in educational and developmental contexts.

## Figures and Tables

**Table 1:** Descriptive statistics of Observational Learning Task and Attention and Concentration Battery.

	*M*	*SD*	Minimum	Maximum
***Observational Learning Task—Phases***				
DP errors	20.71	18.87	0	80
EP repetitions	3.71	7.81	0	57
AP time	8.86	2.52	4.84	17.84
***Observational Learning Task—Errors***				
Perseverative errors	1.91	4.13	0	28
Sequence errors	13.73	14.64	0	68
Side-by-side errors	5.51	7.03	0	37
Illogical errors	0.88	2.63	0	15
Imitative errors	5.78	6.88	0	35
***Attention and Concentration Battery***				
*Simple Reaction Time*				
Correct	27.65	2.57	20	30
RTs	0.42	0.08	0.29	0.60
*Speed and Accuracy*				
Correct	25.79	3.85	9	30
RTs	1.29	0.28	0.87	2.98
*Auditory Recognition*				
Correct	8.85	0.49	6	9
RTs	0.79	0.17	0.52	1.50
*Visual Recognition*				
Correct	7.66	1.77	1	9
RTs	0.58	0.07	0.43	0.70
*Visual–Spatial Recognition*				
Correct	11.60	1.04	6	12
RTs	0.51	0.11	0.32	0.76
*Digit Span*				
Total sum	6.22	1.87	2	11
*Divided Attention*				
Correct	8.28	1.14	4	9
RTs	1.11	0.24	0.65	1.96
*Alternating Attention–Letters*				
Correct	8.50	0.89	4	9
RTs	13.85	16.59	3.82	137.16
*Alternating Attention–Symbols*				
Correct	7.63	1.79	1	9
RTs	21.34	25.54	5.39	207.75

**Table 2:** Partial correlations of Observational Learning Task phases parameters, and Attention and Concentration Battery scores, with age and gender as covariates.

	DP errors	EP Repetitions	AP Times
Simple Reaction Time—Correct	−0.17	−0.21	0.21
Simple Reaction Time—RTs	0.12	0.22	0.01
Speed and Accuracy—Correct	−0.13	−0.59 **	0.005
Speed and Accuracy—RTs	−0.02	0.65 **	0.07
Auditory Recognition—Correct	−0.07	−0.17	0.19
Auditory Recognition—RTs	0.21	0.13	−0.11
Visual Recognition—Correct	−0.31	−0.004	0.13
Visual Recognition—RTs	0.23	0.05	0.008
Visual–Spatial Recognition—Correct	−0.33	−0.67 **	0.09
Visual–Spatial Recognition—RTs	0.13	0.14	0.11
Digit Span—Total sum	−0.47 *	−0.25	0.23
Divided Attention—Correct	−0.009	−0.20	−0.08
Divided Attention—RTs	0.17	0.16	0.06
Alternating Attention–Letters—Correct	−0.008	0.06	0.09
Alternating Attention–Letters—RTs	0.23	0.11	−0.05
Alternating Attention–Symbols—Correct	−0.12	−0.18	0.13
Alternating Attention–Symbols—RTs	0.35	0.26	−0.08

*Note*. DP: Detection Phase; EP: Exercise Phase; AP: Automatization Phase; RTs: Response Times. Values in bold indicate statistical significance. * *p* < 0.05; ** *p* < 0.001.

**Table 3:** Multiple regression analyses examining the contribution of age, gender, and Attention and Concentration Battery scores to Observational Learning Task phases.

	DP Errors		EP Repetitions		AP Times	
Variables	*B*	*t*	*B*	*t*	*B*	*t*
F	2.18 *		4.91 **		1.09	
R^2^	0.46		0.66		0.30	
Age	0.21	1.62	0.07	0.71	−0.31 *	−2.02 *
Gender	0.21	0.76	−0.07	−0.33	−0.15	−0.49
*Simple Reaction Time*						
Correct	−0.09	−0.58	−0.08	−0.62	0.29	1.56
RTs	−0.20	−1.10	−0.08	−0.53	0.08	0.36
*Speed and Accuracy*						
Correct	−0.009	−0.05	−0.03	−0.17	0.02	0.10
RTs	−0.42 *	−2.25 *	0.31 *	2.13 *	0.31	1.45
*Auditory Recognition*						
Correct	0.03	0.26	−0.09	−0.93	0.12	0.94
RTs	0.06	0.42	−0.003	−0.03	−0.21	−1.34
*Visual Recognition*						
Correct	−0.19	−1.12	0.14	1.01	0.06	0.27
RTs	0.15	1.15	−0.14	−1.32	0.11	0.69
*Visual–Spatial Recognition*						
Correct	−0.41 *	−2.04 *	−0.58 **	−3.67 **	0.28	1.22
RTs	−0.04	−0.29	0.04	0.33	0.19	1.18
*Digit Span*						
Total Sum	−0.29	−1.86	−0.08	−0.62	0.07	0.43
*Divided Attention*						
Correct	−0.04	−0.35	−0.03	−0.29	−0.11	−0.79
RTs	0.11	0.96	0.11	1.20	0.06	0.41
*Alternating Attention–Letters*						
Correct	0.09	0.74	0.09	0.94	0.08	0.57
RTs	0.06	0.36	0.04	0.77	−0.07	−0.41
*Alternating Attention–Symbols*						
Correct	−0.14	−1.08	−0.14	−1.39	0.11	0.76
RTs	0.11	0.60	−0.18	−1.27	0.08	0.38

*Note.* DP: Detection Phase; EP: Exercise Phase; AP: Automatization Phase; RTs: Response Times. Values in bold indicate statistical significance. * *p* < 0.05; ** *p* < 0.001.

**Table 4:** Partial correlations of Observational Learning Task errors and Attention and Concentration Battery scores, with age and gender as covariates.

	Perseverative Errors	Sequence Errors	Side-by-Side Errors	Illogical Errors	Imitative Errors
Simple Reaction Time—Correct	−0.13	−0.27	−0.12	−0.16	−0.18
Simple Reaction Time—RTs	0.24	0.16	0.11	0.14	0.18
Speed and Accuracy—Correct	−0.31	−0.36	−0.24	−0.25	−0.46 **
Speed and Accuracy—RTs	0.39	0.29	0.16	0.13	0.30
Auditory Recognition—Correct	−0.13	−0.18	−0.07	0.10	−0.04
Auditory Recognition—RTs	0.25	0.27	0.15	0.19	0.18
Visual Recognition—Correct	−0.08	−0.18	−0.36	−0.26	−0.24
Visual Recognition—RTs	0.18	0.18	0.04	0.12	0.09
Visual–Spatial Recognition—Correct	−0.38	−0.59 **	−0.44 *	−0.36	−0.61 **
Visual–Spatial Recognition—RTs	0.17	0.13	0.16	0.05	0.19
Digit Span—Total sum	−0.36	−0.41	−0.41	−0.28	−0.40
Divided Attention—Correct	−0.12	−0.05	−0.05	0.01	−0.09
Divided Attention—RTs	0.22	0.19	0.13	0.18	0.10
Alternating Attention–Letters—Correct	0.14	0.02	0.03	0.04	0.07
Alternating Attention–Letters—RTs	0.032	0.18	0.23	0.18	0.27
Alternating Attention–Symbols—Correct	−0.29	−0.1	−0.05	−0.002	−0.20
Alternating Attention–Symbols—RTs	0.07	0.42 *	0.38	0.46 **	0.46 **

Note. RTs: Response Times. Values in bold indicate statistical significance. * *p* < 0.05; ** *p* < 0.001.

**Table 5:** Multiple regression analyses examining the contribution of age, gender, and Attention and Concentration Battery scores to Observational Learning Task errors.

	Perseverative Errors		Sequence Errors		Side-by-Side Errors		Illogical Errors		Imitative Errors	
Variables	*B*	*t*	*B*	*t*	*B*	*t*	*B*	*t*	*B*	*t*
F	2.07 *		2.98 **		1.91 *		1.61		3.24 **	
R^2^	0.45		0.54		0.43		0.39		0.56	
Age	0.04	0.29	0.22	1.80	0.24	1.76	0.37 *	2.57 *	0.24	1.98
Gender	0.39	1.39	0.05	0.19	0.009	0.034	−0.18	−0.62	0.09	0.38
*Simple Reaction Time*										
Correct	0.12	0.72	−0.27	−1.82	0.07	0.421	−0.09	−0.56	−0.10	−0.69
RTs	0.005	0.03	−0.26	−1.55	−0.19	−1.01	−0.05	−0.27	−0.20	−1.24
*Speed and Accuracy*										
Correct	0.16	0.78	−0.0006	−0.004	0.21	1.04	−0.14	−0.65	−0.15	−0.82
RTs	2.19	1.01	−0.21	−1.23	−0.009	−0.05	−1.11	−0.58	−0.16	−0.95
*Auditory Recognition*										
Correct	−0.49	−0.41	−0.06	−0.60	0.02	0.17	0.22	1.77	0.07	0.66
RTs	0.10	0.72	0.17	1.34	−0.08	−0.55	−0.01	−0.07	−0.0006	−0.05
*Visual Recognition*										
Correct	0.06	0.32	0.03	0.17	−0.47 *	−2.55 *	−0.26	−1.38	−0.18	−1.11
RTs	−0.03	−0.23	−0.02	−0.17	−0.09	−0.68	0.05	0.39	−0.02	−0.19
*Visual–Spatial Recognition*										
Correct	−0.44 *	−2.16 *	−0.65 **	−3.51 **	−0.51 *	−2.49 *	−0.12	−0.57	−0.55 *	−3.03 *
RTs	0.05	0.34	−0.004	−0.03	0.005	0.03	−0.18	−1.17	0.04	0.35
*Digit Span*										
Total Sum	−0.28	−1.74	−0.17	−1.15	−0.20	−1.27	−0.18	−1.10	−0.17	−1.20
*Divided Attention*										
Correct	−0.05	−0.39	0.01	0.11	−0.03	−0.26	0.05	0.36	0.02	0.18
RTs	0.18	1.52	0.15	1.39	0.05	0.41	0.04	0.32	0.004	0.04
*Alternating Attention–Letters*										
Correct	0.21	1.71	0.08	0.74	0.13	1.08	0.09	0.72	0.16	1.51
RTs	−0.05	−0.33	0.08	0.6	−0.07	−0.44	−0.14	−0.89	0.09	0.64
*Alternating Attention–Symbols*										
Correct	−0.26 *	−2.01 *	−1.18	−1.48	−0.15	−1.16	−0.02	−0.13	−0.23	−1.95
RTs	−0.23	−1.31	0.02	0.12	0.17	0.95	0.42 *	2.23 *	0.09	0.59

*Note.* RTs: Response Times. Values in bold indicate statistical significance. * *p* < 0.05; ** *p* < 0.001.

## Data Availability

The data presented in this study are available on request from the corresponding author due to Department policy.

## Abbreviations

The following abbreviations are used in this manuscript:

DP	Detection Phase
EP	Exercise Phase
AP	Automatization Phase
OBS	Observational Learning Task
RTs	Response Times
